# The synthesis and crystal structure of bis­[3,3-diethyl-1-(phenyl­imino-κ*N*)thio­urea-κ*S*]silver hexa­fluorido­phosphate

**DOI:** 10.1107/S2056989019011824

**Published:** 2019-08-30

**Authors:** Vincent M. Groner, Garrett E. Larson, Yuwei Kan, Mark F. Roll, James G. Moberly, Kristopher V. Waynant

**Affiliations:** a Department of Chemistry, 875 Perimeter Dr. MS 2343 Moscow, ID 83844, USA; b Department of Chemical & Materials Engineering, 875 Perimeter Dr. MS 1021 Moscow, ID 83844, USA

**Keywords:** crystal structure, aryl­azo­thio­formamide, silver, distorted square planar, square-pyramidal, polymeric chain, hydrogen bonding

## Abstract

The structure of the title complex, [Ag(C_11_H_15_N_3_S)_2_]PF_6_, has monoclinic (*P*2_1_/*c*) symmetry, and the silver atom has a distorted square-planar geometry. The coordination complex crystallized from mixing silver hexa­fluorido­phosphate with a concentrated tetra­hydro­furan solution of *N*,*N*-di­ethyl­phenyl­azo­thio­formamide [ATF; systematic name: 3,3-diethyl-1-(phenyl­imino)­thio­urea] under ambient conditions. The resultant coordination complex exhibits a 2:1 ligand-to-metal ratio, with the silver(I) atom having a fourfold AgN_2_S_2_ coordination sphere, with a single PF_6_ counter-ion. In the crystal, however, one sulfur atom from an ATF ligand of a neighboring complex coordinates to the silver atom, with a bond distance of 2.9884 (14) Å. This creates a polymeric zigzag chain propagating along the *c*-axis direction. The chains are linked by C—H⋯F hydrogen bonds, forming slabs parallel to the *ac* plane.

## Abstract 2

The distorted title square-planar silver(I) complex was obtained in very good yield after gentle mixing of solutions of the *N*,*N*-di­ethyl­phenyl­azo­thio­formamide (ATF) ligand with silver hexa­fluorido­phosphate in tetra­hydro­furan. In the crystal, one sulfur atom from an ATF ligand of a neighboring complex coordinates to the silver atom, with a bond distance of 2.9884 (14) Å. This creates a polymeric zigzag chain propagating along the *c*-axis direction.

## Chemical context   

The redox-active azo­thio­formamide (ATF) ligand class was identified as a metal coordinative species over 40 years ago (Bechgaard, 1974 ▸, 1977 ▸). These ligands were found to coord­inate and solvate late transition metal(0) species, particularly Cu, Pd, Pt, and Ni (Nielsen *et al.*, 2007 ▸). Further investigations found that ATF ligands were capable of removing similar late transition metal (Cu or Pd) nanoparticles and catalysts from polymeric materials (Nielsen *et al.*, 2005 ▸, 2006 ▸). As these ligands are redox-active, it was suggested that, during coord­ination, the two ligands singly reduce as the metal oxidizes to (+2) and coordinates in a 2:1 fashion of ligands to metal. This observation was confirmed utilizing computational comparisons of crystal structures from the found species and a copper(I) complex (Johnson *et al.*, 2017 ▸). Those comparisons led to the discovery that ATF ligands stay neutral when mixed with copper(I) salts behaving as 1:1 species in the presence of halide counter-ions and 2:1 species in the presence of non-coordinating counter-ions (such as BF_4_ and PF_6_). The copper(I) halide coordination complexes crystallize out of concentrated THF solution as dimers yet exhibit 1:1 coordination as observed in titration studies. The importance of understanding the variability in the binding phenomena of the various oxidation states in metals can help determine how and in which oxidation state these ligands can coordinate, solvate and remove metals from materials to allow for higher purity. While most trace-metal removal is accomplished with mineral acids, a mild ligand alternative could allow for the removal of metals from acid sensitive materials such as polymers, pharmaceuticals or APIs, or from metals found in electronic waste (e-waste). Silver(I) catalysts and co-catalysts have become increasingly common over the past twenty years, and with silver a precious metal, the potential value of its recycling following synthetic reactions is worthwhile. The investigation of monovalent metals led to this report describing the coordination complex formed when the *N*,*N*-di­ethyl­phenyl­azo­thio­formamide (ATF) ligand is treated with an Ag(I) species containing the non-coordinative counter-ion hexa­fluorido­phosphate in concentrated THF solution.
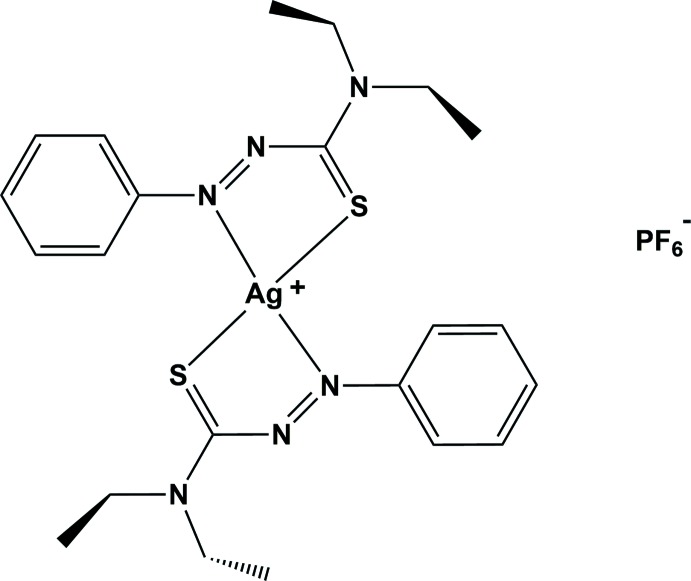


## Structural commentary   

The experiment described herein involved the mixing of AgPF_6_ with a concentrated THF solution of the ATF ligand at room temperature which yielded the title complex in excellent yield (> 95%).

**Figure 1:**
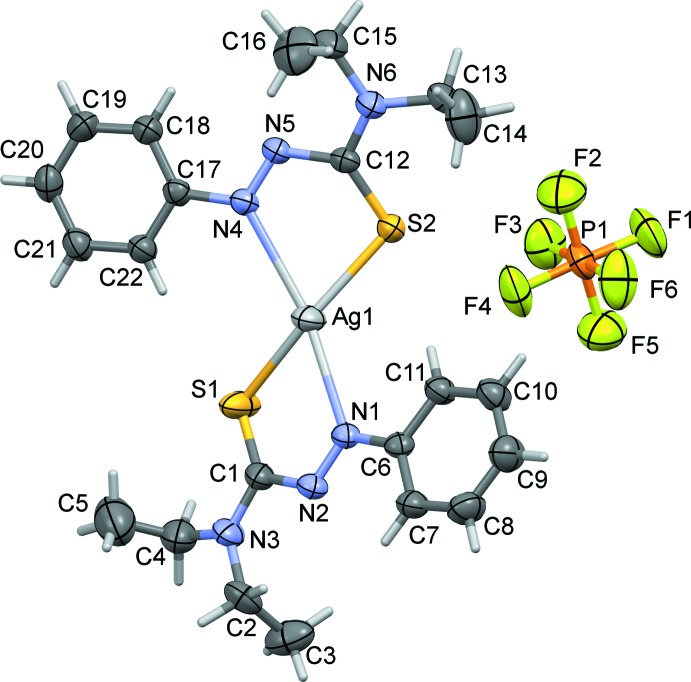
A view of the mol­ecular structure of the asymmetric unit of the title complex, with atom labeling. Displacement ellipsoids are drawn at the 30% probability level.

The mol­ecular structure of the asymmetric unit of the title complex is shown in Fig. 1 ▸. Selected bond lengths and bond angles involving atom Ag1 are given in Table 1 ▸. The silver(I) atom has a distorted square-planar AgN_2_S_2_ coordination geometry with a τ_4_ fourfold parameter of 0.32 (τ_4_ = 1 for a perfect tetra­hedral geometry and 0 for a perfect square-planar geometry. For inter­mediate structures, including trigonal–pyramidal and seesaw, τ_4_ falls within the range of 0 to 1; Yang *et al.*, 2007 ▸). Such distorted square-planar silver complexes, once considered rare have become more common (Chowdhury *et al.*, 2003 ▸; Ino *et al.*, 2000 ▸; Suenaga *et al.*, 2002 ▸; Young & Hanton, 2008 ▸; Pointillart *et al.*, 2008 ▸; Hanton & Young, 2006 ▸). These compounds usually require strengthened bonds through polymeric networks and herein we try to rationalize our structure through a similar network.

**Figure 2:**
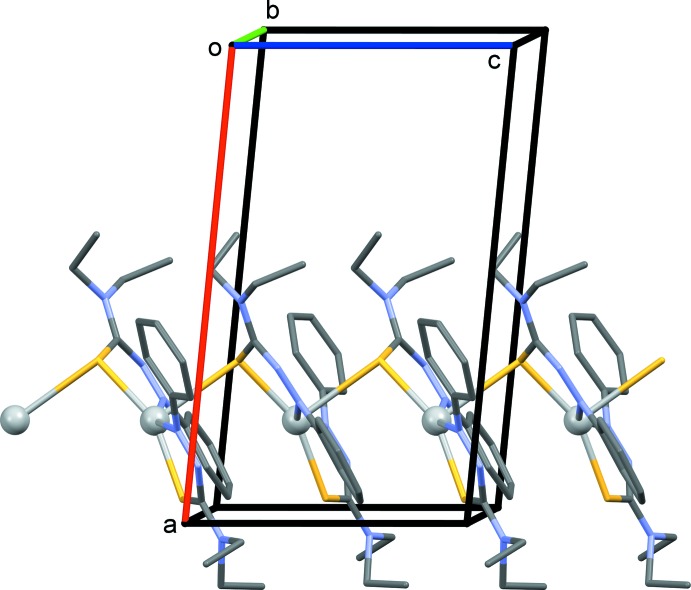
A partial view along the *b* axis of the crystal packing of the title complex. For clarity, the PF_6_ anions and the H atoms have been omitted.

The crystal structure of the ligand ATF has been reported by Johnson *et al.* (2017 ▸). The ATF ligand–bond distances in the title complex match more closely to the neutral species than the singly reduced ligand as the presence of a PF_6_ counter-ion suggests monovalent oxidation of silver. Although the asymmetric unit suggests the 2:1 binding species with two S—Ag and two N—Ag bonds, the N4—Ag1 bond is lengthened in comparison with previously mentioned complexes (Johnson *et al.*, 2017 ▸). This lengthening has influenced the packing structure of the crystal to allow for an adjacent ATF ligand to inter­act with the silver atom at a bond distance Ag1⋯S2^i^ of 2.9884 (14) Å, producing a polymeric zigzag chain (Fig. 2 ▸ and Table 1 ▸). If atom Ag1 is now considered to be fivefold AgN_2_S_3_ coordinate it has a perfect square-pyramidal geometry with a τ_5_ fivefold parameter of 0.04 (τ_5_ = 1 for perfect trigonal–pyramidal geometry and 0 for perfect square-pyramidal geometry; Addison *et al.*, 1984 ▸). Sulfur atom S1 is involved in an intra­molecular C—H⋯S hydrogen bond (Fig. 1 ▸ and Table 2 ▸).

The bond distances for ATF ligand complexes were compared to computationally modeled neutral and singly reduced ATF species as to ascertain the absolute oxidation state of the ligands (Johnson *et al.*, 2017 ▸). The computationally compared neutral ligand necessitated rotation at 1.33 kcal mol^−1^ to give a transition state containing the planar 1,4-heterodiene motif while the computationally calculated singly reduced ATF ligand flattens to adopt the binding motif. Table 3 ▸ provides comparative bond distances for these species to known bis-bidentate ATF copper(I), copper(II), and palladium (II) species that are found as distorted tetra­hedral conformations and square-planar nickel(II) and platinum (II) species (Nielsen *et al.*, 2007 ▸; Johnson *et al.*, 2017 ▸).

Also, to note, is that repeated attempts to create the silver(I) tetra­fluoro­borate variation were unsuccessful. UV–Vis absorbance in aceto­nitrile displayed no photophysical properties or effects. The melting point of the complex was found to occur at 329 K, which is similar to the melting point of 325 K for the ligand, further suggesting the weak binding inter­action.

## Supra­molecular features   

**Figure 3:**
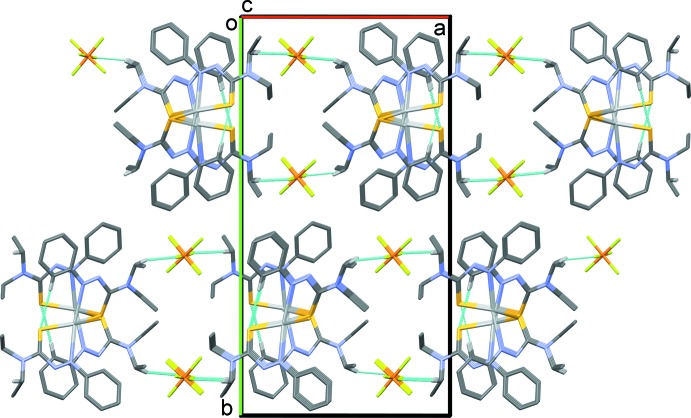
A view along the *c* axis of the crystal packing of the title complex. The C—H⋯S and C—H⋯F hydrogen bonds are shown as dashed lines. For clarity, only the H atoms involved in hydrogen bonding have been included.

In the crystal, the polymeric zigzag chains that propagate along the *c*-axis direction, are linked by C—H⋯F hydrogen bonds, forming slabs parallel to the *ac* plane (Table 3 ▸ and Fig. 3 ▸).

The two ligands in the title complex crystal are asymmetric in regard to their respective distances to the silver atom from the coordinating sulfur and nitro­gen atoms of each ligand and asymmetric in the geometries of the two diethyl thio­formamide units on each ligand (Figs. 1 ▸ and 2 ▸, and Table 1 ▸). It is proposed that the inter­action between the adjacent sulfur atom to the bis-coordinated silver, as shown in Fig. 2 ▸, provides the asymmetry in the binding inter­action as the sulfur of the second ATF (that does not conjugate to a bridging silver atom) is slightly closer to its silver atom than the ligand that contains the polymeric sulfur bridge. The packing structure also displays an alternating coordination throughout the crystalline lattice connecting silver atoms to sulfurs. The distorted square-planar structure is rare in silver(I) systems and it is suggested that the inter­connecting sulfur atom ladder-like chain structure strengthens the framework (Shin *et al.*, 2009 ▸). Secondly, the second bound ATF ligand displays both ethyl groups in the diethyl group of the thio­formamide facing in the same direction instead of opposite directions as seen in the crystal structure of the ligand (Johnson *et al.*, 2017 ▸), and thus a higher energy kinetic state (Shin *et al.*, 2009 ▸).

It is suggested that the large PF_6_ counter-ions inhibit the rotation of the second ethyl group so as to allow for more space. Counter-anion influence for silver coordination complexes has been seen in other systems (Zhao *et al.*, 2012 ▸; Huang *et al.*, 2008 ▸).

## Synthesis and crystallization   

**Figure 4:**
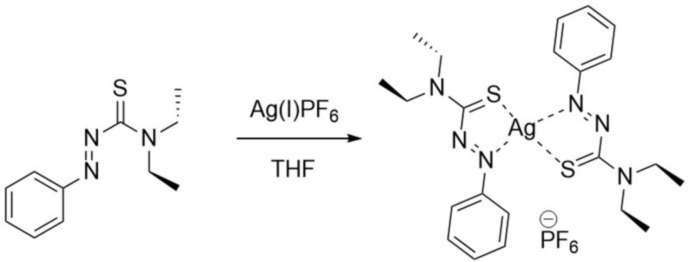
The reaction scheme for the synthesis of the title complex.

The reaction scheme for the synthesis of the title complex is given in Fig. 4 ▸. Silver hexa­fluorido­phosphate (29.2 mg, 0.115 mmol) was added to a solution of *N*,*N*-di­ethyl­phenyl­azo­thio­formamide (ATF; 51 mg, 0.230 mmol) in 3 ml of tetra­hydro­furan and the mixture immediately darkened from light orange to a burgundy in color. The solution was concentrated *via* rotary evaporation and the solid obtained was purified by multiple cold hexane washes to remove any excess ligand, providing 75.0 mg (93.6% yield) of a burgundy solid. For crystallization, 35 mg of the solid were dissolved in 2 ml of THF and allowed to slowly concentrate over two days, yielding dark-brown needle-like crystals upon deca­ntation (m.p. 329 K). Further evaporation gave a burgundy solid. ^1^H NMR (300MHz, Chloro­form-*d*) δ 7.95–7.85 (m, 2H), 7.70–7.48 (*m*, 3H), 7.28 (*s*, 7H), 4.30–4.16 (*m*, 2H), 4.07 (*q*, *J* = 7.2Hz, 2H), 1.55 (*t*, *J* = 7.1Hz, 3H), 1.38 (*t*, *J* = 7.2Hz, 3H); ^13^C NMR (75MHz, CDCl3) δ 151.32, 136.86, 130.93, 126.24, 100.85, 52.01, 48.87, 15.53, 11.98.

## Refinement   

Crystal data, data collection and structure refinement details are summarized in Table 4 ▸. The C-bound H-atoms were included in calculated positions and refined as riding on the parent C atom: C—H = 0.93–0.97 Å with *U*_iso_(H) = 1.5*U*_eq_(C-meth­yl) and 1.2*U*_eq_(C) for other H-atoms.

## Supplementary Material

Crystal structure: contains datablock(s) Global, I. DOI: 10.1107/S2056989019011824/su5511sup1.cif

Structure factors: contains datablock(s) I. DOI: 10.1107/S2056989019011824/su5511Isup2.hkl

CCDC reference: 1949718

Additional supporting information:  crystallographic information; 3D view; checkCIF report

## Figures and Tables

**Table 1:** Selected geometric parameters (Å, °)

Ag1—S1	2.4280 (14)	Ag1—N1	2.632 (3)
Ag1—S2	2.4500 (12)	Ag1—N4	2.671 (3)
Ag1—S2^i^	2.9884 (14)		
			
S1—Ag1—S2	156.32 (6)	N1—Ag1—N4	159.01 (10)
S1—Ag1—N1	71.07 (7)	S2—Ag1—N4	71.38 (7)
S1—Ag1—N4	109.01 (7)	S2—Ag1—N1	117.39 (7)

Symmetry code: (i) 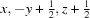.

**Table 2:** Hydrogen-bond geometry (Å, °)

*D*—H⋯*A*	*D*—H	H⋯*A*	*D*⋯*A*	*D*—H⋯*A*
C22—H22⋯S1	0.93	2.78	3.690 (5)	167
C2—H2*B*⋯F1^ii^	0.97	2.52	3.435 (6)	158
C15—H15*A*⋯F4^iii^	0.97	2.46	3.398 (6)	164

Symmetry codes: (ii) 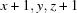; (iii) 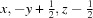.

**Table 3:** Bond lengths (Å) and characteristic geometries of related ATF mono- and divalent metal complexes CSD = Cambridge Structural Database (Groom *et al.*, 2016 ▸); DSP = distorted square-planar; DT = distorted tetra­hedral.

Metal	*M*—N	N=N	N—C	C=S	*M*—S	*M*⋯S	Structure	CSD refcode
Ag^I*a*^	2.632 (N1)	1.242 (N1=N2)	1.442 (N2—C1)	1.656 (C1=S1)	2.428 (S1)		DSP	
Ag^I*a*^	2.671 (N4)	1.233 (N4=N5)	1.424 (N5—C12)	1.685 (C12=S2)	2.450 (S2)	2.988 (S2^i^)		
Cu^I*b*^	1.986 / 2.005	1.265 / 1.263	1.417 / 1.429	1.691 / 1.689	2.280 / 2.275		DT	WELGAY
Cu^I*b*^	1.994 / 1.985	1.272 / 1.273	1.427 / 1.428	1.701 / 1.696	2.280 / 2.284		DT	WELFUR
								
Cu^II*c*^	1.922	1.323	1.371	1.722	2.276		DT	KEYBIA
Pd^II*c*^	1.993	1.339	1.34	1.741	2.293		DT	KEYBOG
Pt^II*c*^	1.964	1.349	1.326	1.742	2.293		DSP	KEXCAT
Ni^II*c*^	1.873	1.336	1.358	1.721	2.209		DSP	NIEPZF01
								
ATF (crystal)^*b*^		1.244	1.44	1.662				WELFOL
ATF TS (modeled)		1.254	1.448	1.671				
ATF SOMO (modeled)		1.329	1.357	1.72				

Notes: (*a*) This study; (*b*) Johnson *et al.* (2017 ▸); (*c*) Nielsen *et al.* (2007 ▸). Symmetry code: (i) *x*, −*y* + 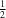, *z* + 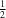.

**Table 4:** Experimental details

Crystal data	
Chemical formula	[Ag(C_11_H_15_N_3_S)_2_]PF_6_
*M*_r_	695.48
Crystal system, space group	Monoclinic, *P*2_1_/*c*
Temperature (K)	296
*a*, *b*, *c* (Å)	13.827 (2), 26.243 (4), 8.1218 (15)
β (°)	95.678 (12)
*V* (Å^3^)	2932.6 (9)
*Z*	4
Radiation type	Mo *K*α
μ (mm^−1^)	0.95
Crystal size (mm)	0.50 × 0.10 × 0.02

Data collection	
Diffractometer	Bruker *SMART* APEXII area detector
Absorption correction	Multi-scan (*SADABS*; Bruker, 2003 ▸)
*T*_min_, *T*_max_	0.867, 1.000
No. of measured, independent and observed [*I* > 2σ(*I*)] reflections	46111, 5118, 2829
*R*_int_	0.084
(sin θ/λ)_max_ (Å^−1^)	0.594

Refinement	
*R*[*F* ^2^ > 2σ(*F* ^2^)], *wR*(*F* ^2^), *S*	0.039, 0.101, 1.00
No. of reflections	5118
No. of parameters	347
H-atom treatment	H-atom parameters constrained
Δρ_max_, Δρ_min_ (e Å^−3^)	0.41, −0.33

Computer programs: *APEX2* and *SAINT* (Bruker, 2003 ▸), *SHELXT* (Sheldrick, 2015*a* ▸), *SHELXL2018* (Sheldrick, 2015*b* ▸), *OLEX2* (Dolomanov *et al.*, 2009 ▸) and *Mercury* (Macrae *et al.*, 2008 ▸).

## supplementary crystallographic information

### Crystal data

**Table:**

[Ag(C_11_H_15_N_3_S)_2_]PF_6_	*F*(000) = 1408
*M**_r_* = 695.48	*D*_x_ = 1.575 Mg m^−^^3^
Monoclinic, *P*2_1_/*c*	Mo *K*α radiation, λ = 0.71073 Å
*a* = 13.827 (2) Å	Cell parameters from 4249 reflections
*b* = 26.243 (4) Å	θ = 2.6–18.2°
*c* = 8.1218 (15) Å	µ = 0.95 mm^−^^1^
β = 95.678 (12)°	*T* = 296 K
*V* = 2932.6 (9) Å^3^	Needle, dark_brown
*Z* = 4	0.50 × 0.10 × 0.02 mm

### Data collection

**Table:**

Bruker SMART APEXII area detector diffractometer	5118 independent reflections
Radiation source: microfocus sealed X-ray tube, Incoatec Iµs	2829 reflections with *I* > 2σ(*I*)
Mirror optics monochromator	*R*_int_ = 0.084
ω and φ scans	θ_max_ = 25.0°, θ_min_ = 1.7°
Absorption correction: multi-scan (SADABS; Bruker, 2003)	*h* = −16→16
*T*_min_ = 0.867, *T*_max_ = 1.000	*k* = −30→30
46111 measured reflections	*l* = −9→9

### Refinement

**Table:**

Refinement on *F*^2^	Primary atom site location: dual
Least-squares matrix: full	Secondary atom site location: difference Fourier map
*R*[*F*^2^ > 2σ(*F*^2^)] = 0.039	Hydrogen site location: inferred from neighbouring sites
*wR*(*F*^2^) = 0.101	H-atom parameters constrained
*S* = 1.00	*w* = 1/[σ^2^(*F*_o_^2^) + (0.0336*P*)^2^ + 2.2376*P*] where *P* = (*F*_o_^2^ + 2*F*_c_^2^)/3
5118 reflections	(Δ/σ)_max_ < 0.001
347 parameters	Δρ_max_ = 0.41 e Å^−^^3^
0 restraints	Δρ_min_ = −0.33 e Å^−^^3^

### Special details

**Table:**

Geometry. All esds (except the esd in the dihedral angle between two l.s. planes) are estimated using the full covariance matrix. The cell esds are taken into account individually in the estimation of esds in distances, angles and torsion angles; correlations between esds in cell parameters are only used when they are defined by crystal symmetry. An approximate (isotropic) treatment of cell esds is used for estimating esds involving l.s. planes.

### Fractional atomic coordinates and isotropic or equivalent isotropic displacement parameters (Å^2^)

**Table:**

	*x*	*y*	*z*	*U*_iso_*/*U*_eq_	
Ag1	0.79300 (3)	0.26718 (2)	0.33206 (6)	0.08610 (18)	
S1	0.95699 (10)	0.28141 (5)	0.4601 (3)	0.1154 (6)	
S2	0.66730 (9)	0.24699 (4)	0.10744 (14)	0.0641 (3)	
N1	0.8100 (2)	0.36299 (13)	0.4266 (4)	0.0565 (9)	
N2	0.8839 (3)	0.37758 (13)	0.5130 (4)	0.0625 (9)	
N3	1.0378 (3)	0.35913 (15)	0.6217 (5)	0.0857 (12)	
N4	0.7521 (2)	0.16786 (12)	0.3449 (4)	0.0547 (9)	
N5	0.6803 (2)	0.15201 (13)	0.2576 (4)	0.0585 (9)	
N6	0.5405 (3)	0.17148 (14)	0.1021 (5)	0.0708 (10)	
C1	0.9604 (3)	0.34016 (17)	0.5348 (6)	0.0699 (13)	
C2	1.0426 (4)	0.4114 (2)	0.6960 (6)	0.0889 (16)	
H2A	0.977716	0.422803	0.713474	0.107*	
H2B	1.081255	0.410568	0.802377	0.107*	
C3	1.0867 (5)	0.4471 (2)	0.5841 (8)	0.127 (2)	
H3A	1.153509	0.438117	0.577860	0.190*	
H3B	1.083027	0.481205	0.626125	0.190*	
H3C	1.052130	0.445301	0.475804	0.190*	
C4	1.1360 (4)	0.3288 (2)	0.6314 (9)	0.115 (2)	
H4A	1.138731	0.308004	0.533130	0.139*	
H4B	1.190720	0.352053	0.639940	0.139*	
C5	1.1387 (6)	0.2978 (3)	0.7730 (10)	0.178 (3)	
H5A	1.091958	0.270819	0.754251	0.266*	
H5B	1.123377	0.317915	0.865694	0.266*	
H5C	1.202479	0.283479	0.795836	0.266*	
C6	0.7355 (3)	0.40000 (15)	0.3992 (5)	0.0550 (11)	
C7	0.7384 (4)	0.44803 (18)	0.4695 (6)	0.0773 (14)	
H7	0.791760	0.457888	0.541257	0.093*	
C8	0.6625 (4)	0.4811 (2)	0.4331 (8)	0.1002 (18)	
H8	0.664225	0.513353	0.480777	0.120*	
C9	0.5837 (4)	0.4667 (2)	0.3264 (7)	0.0987 (18)	
H9	0.532637	0.489326	0.301544	0.118*	
C10	0.5803 (4)	0.4192 (2)	0.2566 (6)	0.0852 (15)	
H10	0.526856	0.409577	0.184869	0.102*	
C11	0.6561 (3)	0.38565 (18)	0.2927 (5)	0.0679 (12)	
H11	0.653846	0.353356	0.245253	0.081*	
C12	0.6259 (3)	0.18962 (15)	0.1621 (5)	0.0547 (11)	
C13	0.4753 (4)	0.2005 (2)	−0.0208 (6)	0.0875 (16)	
H13A	0.514610	0.221663	−0.085952	0.105*	
H13B	0.439811	0.176640	−0.095411	0.105*	
C14	0.4051 (5)	0.2331 (3)	0.0560 (9)	0.141 (3)	
H14A	0.360649	0.248003	−0.028846	0.212*	
H14B	0.439459	0.259682	0.118619	0.212*	
H14C	0.369689	0.212916	0.128141	0.212*	
C15	0.5034 (4)	0.1206 (2)	0.1519 (7)	0.0886 (16)	
H15A	0.453556	0.108845	0.067941	0.106*	
H15B	0.556110	0.096067	0.159018	0.106*	
C16	0.4625 (5)	0.1234 (3)	0.3130 (8)	0.140 (3)	
H16A	0.404716	0.143881	0.301966	0.210*	
H16B	0.509426	0.138431	0.393629	0.210*	
H16C	0.446866	0.089702	0.347956	0.210*	
C17	0.8103 (3)	0.12975 (16)	0.4313 (5)	0.0554 (11)	
C18	0.7864 (4)	0.07848 (17)	0.4280 (6)	0.0715 (13)	
H18	0.729062	0.067045	0.370421	0.086*	
C19	0.8499 (4)	0.04485 (19)	0.5122 (6)	0.0878 (16)	
H19	0.835162	0.010260	0.511021	0.105*	
C20	0.9342 (4)	0.0616 (2)	0.5977 (6)	0.0809 (15)	
H20	0.976496	0.038311	0.653103	0.097*	
C21	0.9566 (3)	0.1123 (2)	0.6018 (6)	0.0770 (14)	
H21	1.013913	0.123608	0.660001	0.092*	
C22	0.8938 (3)	0.14649 (17)	0.5194 (5)	0.0663 (12)	
H22	0.908173	0.181116	0.523478	0.080*	
P1	0.26919 (10)	0.40202 (6)	0.18758 (18)	0.0830 (4)	
F1	0.2325 (3)	0.40775 (17)	0.0024 (4)	0.1623 (16)	
F2	0.1982 (3)	0.35750 (18)	0.2091 (6)	0.1776 (18)	
F3	0.3494 (3)	0.36342 (14)	0.1466 (4)	0.1379 (13)	
F4	0.3058 (3)	0.39833 (15)	0.3751 (4)	0.1415 (14)	
F5	0.3402 (3)	0.44721 (16)	0.1657 (6)	0.1590 (15)	
F6	0.1902 (3)	0.44166 (17)	0.2304 (5)	0.1541 (16)	

### Atomic displacement parameters (Å^2^)

**Table:**

	*U*^11^	*U*^22^	*U*^33^	*U*^12^	*U*^13^	*U*^23^
Ag1	0.0721 (3)	0.0667 (3)	0.1133 (4)	−0.0152 (2)	−0.0220 (2)	−0.0073 (2)
S1	0.0699 (9)	0.0616 (8)	0.2041 (18)	−0.0018 (7)	−0.0389 (10)	−0.0147 (9)
S2	0.0734 (8)	0.0597 (7)	0.0575 (7)	−0.0086 (6)	−0.0027 (6)	0.0016 (5)
N1	0.051 (2)	0.058 (2)	0.060 (2)	−0.0100 (18)	0.0010 (18)	0.0033 (18)
N2	0.052 (2)	0.065 (2)	0.068 (2)	−0.0124 (19)	−0.003 (2)	−0.0017 (19)
N3	0.060 (3)	0.075 (3)	0.115 (3)	−0.010 (2)	−0.028 (2)	0.002 (2)
N4	0.050 (2)	0.054 (2)	0.059 (2)	−0.0007 (17)	0.0017 (19)	−0.0089 (18)
N5	0.054 (2)	0.059 (2)	0.060 (2)	−0.0077 (18)	−0.0050 (19)	−0.0011 (18)
N6	0.062 (2)	0.073 (3)	0.074 (3)	−0.013 (2)	−0.009 (2)	0.005 (2)
C1	0.057 (3)	0.064 (3)	0.085 (3)	−0.009 (2)	−0.011 (3)	0.005 (3)
C2	0.080 (4)	0.117 (5)	0.067 (3)	−0.012 (3)	−0.009 (3)	−0.023 (3)
C3	0.152 (6)	0.098 (5)	0.138 (6)	−0.036 (4)	0.050 (5)	−0.012 (4)
C4	0.106 (5)	0.098 (4)	0.135 (6)	−0.022 (4)	−0.025 (4)	0.019 (4)
C5	0.151 (7)	0.210 (9)	0.162 (8)	−0.025 (7)	−0.029 (6)	0.050 (7)
C6	0.048 (3)	0.056 (3)	0.062 (3)	−0.008 (2)	0.008 (2)	0.000 (2)
C7	0.068 (3)	0.065 (3)	0.095 (4)	−0.010 (3)	−0.011 (3)	−0.006 (3)
C8	0.099 (4)	0.064 (3)	0.134 (5)	0.003 (3)	−0.003 (4)	−0.013 (3)
C9	0.080 (4)	0.086 (4)	0.127 (5)	0.016 (3)	−0.007 (4)	−0.005 (4)
C10	0.064 (3)	0.095 (4)	0.092 (4)	0.006 (3)	−0.012 (3)	−0.012 (3)
C11	0.057 (3)	0.070 (3)	0.074 (3)	−0.001 (2)	−0.006 (3)	−0.009 (2)
C12	0.053 (3)	0.058 (3)	0.052 (3)	−0.008 (2)	0.004 (2)	−0.009 (2)
C13	0.073 (4)	0.097 (4)	0.086 (4)	−0.014 (3)	−0.023 (3)	0.001 (3)
C14	0.111 (5)	0.181 (7)	0.131 (6)	0.064 (5)	0.006 (4)	0.024 (5)
C15	0.077 (4)	0.094 (4)	0.091 (4)	−0.023 (3)	−0.013 (3)	−0.005 (3)
C16	0.148 (6)	0.164 (7)	0.117 (6)	−0.017 (5)	0.056 (5)	0.029 (5)
C17	0.057 (3)	0.055 (3)	0.053 (3)	0.003 (2)	0.002 (2)	−0.008 (2)
C18	0.080 (3)	0.062 (3)	0.068 (3)	−0.004 (3)	−0.010 (3)	−0.001 (3)
C19	0.115 (5)	0.058 (3)	0.086 (4)	−0.004 (3)	−0.011 (3)	0.004 (3)
C20	0.092 (4)	0.082 (4)	0.067 (3)	0.023 (3)	−0.001 (3)	0.007 (3)
C21	0.066 (3)	0.086 (4)	0.075 (3)	0.007 (3)	−0.009 (3)	0.000 (3)
C22	0.063 (3)	0.062 (3)	0.071 (3)	0.000 (2)	−0.007 (3)	−0.005 (2)
P1	0.0583 (8)	0.1078 (11)	0.0787 (10)	0.0101 (9)	−0.0138 (7)	0.0042 (8)
F1	0.172 (4)	0.209 (4)	0.092 (3)	0.006 (3)	−0.057 (2)	0.006 (3)
F2	0.133 (3)	0.167 (4)	0.229 (5)	−0.052 (3)	−0.002 (3)	0.046 (3)
F3	0.132 (3)	0.149 (3)	0.134 (3)	0.053 (3)	0.018 (2)	−0.011 (2)
F4	0.155 (3)	0.174 (4)	0.086 (2)	0.061 (3)	−0.037 (2)	−0.007 (2)
F5	0.110 (3)	0.145 (3)	0.219 (4)	−0.028 (3)	0.001 (3)	0.015 (3)
F6	0.120 (3)	0.192 (4)	0.153 (3)	0.082 (3)	0.030 (2)	0.036 (3)

### Geometric parameters (Å, º)

**Table:**

Ag1—S1	2.4280 (14)	C8—H8	0.9300
Ag1—S2	2.4500 (12)	C9—C10	1.367 (7)
Ag1—S2^i^	2.9884 (14)	C9—H9	0.9300
Ag1—N1	2.632 (3)	C10—C11	1.378 (6)
Ag1—N4	2.671 (3)	C10—H10	0.9300
S1—C1	1.656 (5)	C11—H11	0.9300
S2—C12	1.685 (4)	C13—C14	1.478 (7)
N1—N2	1.242 (4)	C13—H13A	0.9700
N1—C6	1.417 (5)	C13—H13B	0.9700
N2—C1	1.442 (5)	C14—H14A	0.9600
N3—C1	1.320 (5)	C14—H14B	0.9600
N3—C2	1.497 (6)	C14—H14C	0.9600
N3—C4	1.569 (7)	C15—C16	1.477 (7)
N4—N5	1.233 (4)	C15—H15A	0.9700
N4—C17	1.424 (5)	C15—H15B	0.9700
N5—C12	1.424 (5)	C16—H16A	0.9600
N6—C12	1.321 (5)	C16—H16B	0.9600
N6—C13	1.487 (6)	C16—H16C	0.9600
N6—C15	1.501 (6)	C17—C22	1.370 (5)
C2—C3	1.479 (7)	C17—C18	1.385 (6)
C2—H2A	0.9700	C18—C19	1.377 (6)
C2—H2B	0.9700	C18—H18	0.9300
C3—H3A	0.9600	C19—C20	1.370 (7)
C3—H3B	0.9600	C19—H19	0.9300
C3—H3C	0.9600	C20—C21	1.366 (6)
C4—C5	1.406 (8)	C20—H20	0.9300
C4—H4A	0.9700	C21—C22	1.376 (6)
C4—H4B	0.9700	C21—H21	0.9300
C5—H5A	0.9600	C22—H22	0.9300
C5—H5B	0.9600	P1—F1	1.546 (3)
C5—H5C	0.9600	P1—F2	1.547 (4)
C6—C11	1.381 (5)	P1—F4	1.560 (3)
C6—C7	1.383 (6)	P1—F5	1.561 (4)
C7—C8	1.372 (7)	P1—F3	1.562 (3)
C7—H7	0.9300	P1—F6	1.572 (4)
C8—C9	1.376 (7)		
			
S1—Ag1—S2	156.32 (6)	C10—C11—H11	120.0
S1—Ag1—N1	71.07 (7)	C6—C11—H11	120.0
S1—Ag1—N4	109.01 (7)	N6—C12—N5	110.9 (4)
N1—Ag1—N4	159.01 (10)	N6—C12—S2	122.7 (3)
S2—Ag1—N4	71.38 (7)	N5—C12—S2	126.0 (3)
S2—Ag1—N1	117.39 (7)	C14—C13—N6	113.1 (5)
C1—S1—Ag1	106.96 (17)	C14—C13—H13A	109.0
C12—S2—Ag1	103.39 (15)	N6—C13—H13A	109.0
N2—N1—C6	115.0 (3)	C14—C13—H13B	109.0
N2—N1—Ag1	120.3 (3)	N6—C13—H13B	109.0
C6—N1—Ag1	124.5 (3)	H13A—C13—H13B	107.8
N1—N2—C1	114.3 (4)	C13—C14—H14A	109.5
C1—N3—C2	124.2 (4)	C13—C14—H14B	109.5
C1—N3—C4	119.1 (4)	H14A—C14—H14B	109.5
C2—N3—C4	116.2 (4)	C13—C14—H14C	109.5
N5—N4—C17	115.4 (3)	H14A—C14—H14C	109.5
N4—N5—C12	115.6 (3)	H14B—C14—H14C	109.5
C12—N6—C13	121.5 (4)	C16—C15—N6	111.4 (5)
C12—N6—C15	122.6 (4)	C16—C15—H15A	109.3
C13—N6—C15	115.8 (4)	N6—C15—H15A	109.3
N3—C1—N2	110.8 (4)	C16—C15—H15B	109.3
N3—C1—S1	122.6 (4)	N6—C15—H15B	109.3
N2—C1—S1	126.5 (3)	H15A—C15—H15B	108.0
C3—C2—N3	109.8 (4)	C15—C16—H16A	109.5
C3—C2—H2A	109.7	C15—C16—H16B	109.5
N3—C2—H2A	109.7	H16A—C16—H16B	109.5
C3—C2—H2B	109.7	C15—C16—H16C	109.5
N3—C2—H2B	109.7	H16A—C16—H16C	109.5
H2A—C2—H2B	108.2	H16B—C16—H16C	109.5
C2—C3—H3A	109.5	C22—C17—C18	120.6 (4)
C2—C3—H3B	109.5	C22—C17—N4	116.0 (4)
H3A—C3—H3B	109.5	C18—C17—N4	123.4 (4)
C2—C3—H3C	109.5	C19—C18—C17	118.3 (4)
H3A—C3—H3C	109.5	C19—C18—H18	120.8
H3B—C3—H3C	109.5	C17—C18—H18	120.8
C5—C4—N3	106.7 (6)	C20—C19—C18	121.0 (5)
C5—C4—H4A	110.4	C20—C19—H19	119.5
N3—C4—H4A	110.4	C18—C19—H19	119.5
C5—C4—H4B	110.4	C21—C20—C19	120.3 (5)
N3—C4—H4B	110.4	C21—C20—H20	119.8
H4A—C4—H4B	108.6	C19—C20—H20	119.8
C4—C5—H5A	109.5	C20—C21—C22	119.5 (5)
C4—C5—H5B	109.5	C20—C21—H21	120.2
H5A—C5—H5B	109.5	C22—C21—H21	120.2
C4—C5—H5C	109.5	C17—C22—C21	120.3 (4)
H5A—C5—H5C	109.5	C17—C22—H22	119.9
H5B—C5—H5C	109.5	C21—C22—H22	119.9
C11—C6—C7	119.8 (4)	F1—P1—F2	91.9 (3)
C11—C6—N1	115.6 (4)	F1—P1—F4	178.0 (2)
C7—C6—N1	124.6 (4)	F2—P1—F4	89.5 (3)
C8—C7—C6	119.8 (5)	F1—P1—F5	88.0 (2)
C8—C7—H7	120.1	F2—P1—F5	179.6 (3)
C6—C7—H7	120.1	F4—P1—F5	90.7 (2)
C7—C8—C9	120.2 (5)	F1—P1—F3	91.6 (2)
C7—C8—H8	119.9	F2—P1—F3	90.3 (3)
C9—C8—H8	119.9	F4—P1—F3	89.9 (2)
C10—C9—C8	120.3 (5)	F5—P1—F3	90.0 (2)
C10—C9—H9	119.9	F1—P1—F6	89.0 (2)
C8—C9—H9	119.9	F2—P1—F6	90.7 (3)
C9—C10—C11	120.0 (5)	F4—P1—F6	89.5 (2)
C9—C10—H10	120.0	F5—P1—F6	88.9 (2)
C11—C10—H10	120.0	F3—P1—F6	178.8 (3)
C10—C11—C6	119.9 (4)		
			
C6—N1—N2—C1	−177.8 (3)	C7—C6—C11—C10	0.1 (7)
Ag1—N1—N2—C1	7.9 (5)	N1—C6—C11—C10	−179.3 (4)
C17—N4—N5—C12	−175.7 (3)	C13—N6—C12—N5	−171.3 (4)
C2—N3—C1—N2	3.5 (7)	C15—N6—C12—N5	8.1 (6)
C4—N3—C1—N2	−168.4 (4)	C13—N6—C12—S2	1.0 (6)
C2—N3—C1—S1	−177.9 (4)	C15—N6—C12—S2	−179.5 (4)
C4—N3—C1—S1	10.2 (7)	N4—N5—C12—N6	−166.2 (4)
N1—N2—C1—N3	177.3 (4)	N4—N5—C12—S2	21.7 (5)
N1—N2—C1—S1	−1.3 (6)	Ag1—S2—C12—N6	160.0 (3)
Ag1—S1—C1—N3	175.7 (4)	Ag1—S2—C12—N5	−28.8 (4)
Ag1—S1—C1—N2	−5.9 (5)	C12—N6—C13—C14	−92.2 (6)
C1—N3—C2—C3	−96.0 (6)	C15—N6—C13—C14	88.3 (6)
C4—N3—C2—C3	76.1 (6)	C12—N6—C15—C16	80.1 (6)
C1—N3—C4—C5	−92.4 (7)	C13—N6—C15—C16	−100.4 (5)
C2—N3—C4—C5	95.1 (6)	N5—N4—C17—C22	175.6 (4)
N2—N1—C6—C11	175.1 (4)	N5—N4—C17—C18	−4.0 (6)
Ag1—N1—C6—C11	−10.9 (5)	C22—C17—C18—C19	−1.5 (7)
N2—N1—C6—C7	−4.3 (6)	N4—C17—C18—C19	178.0 (4)
Ag1—N1—C6—C7	169.7 (3)	C17—C18—C19—C20	0.3 (8)
C11—C6—C7—C8	0.1 (7)	C18—C19—C20—C21	0.5 (8)
N1—C6—C7—C8	179.4 (5)	C19—C20—C21—C22	−0.1 (8)
C6—C7—C8—C9	−0.3 (9)	C18—C17—C22—C21	2.0 (7)
C7—C8—C9—C10	0.4 (9)	N4—C17—C22—C21	−177.6 (4)
C8—C9—C10—C11	−0.3 (9)	C20—C21—C22—C17	−1.2 (7)
C9—C10—C11—C6	0.0 (8)		

Symmetry code: (i) *x*, −*y*+1/2, *z*+1/2.

### Hydrogen-bond geometry (Å, º)

**Table:**

*D*—H···*A*	*D*—H	H···*A*	*D*···*A*	*D*—H···*A*
C22—H22···S1	0.93	2.78	3.690 (5)	167
C2—H2*B*···F1^ii^	0.97	2.52	3.435 (6)	158
C15—H15*A*···F4^iii^	0.97	2.46	3.398 (6)	164

Symmetry codes: (ii) *x*+1, *y*, *z*+1; (iii) *x*, −*y*+1/2, *z*−1/2.
